# Influence of Oxygen Flow Rate on Channel Width Dependent Electrical Properties of Indium Gallium Zinc Oxide Thin-Film Transistors

**DOI:** 10.3390/nano10122357

**Published:** 2020-11-27

**Authors:** Gwomei Wu, Anup K. Sahoo

**Affiliations:** Institute of Electro-Optical Engineering, Chang Gung University, Chang Gung Memorial Hospital, Taoyuan 333, Taiwan; anup140387@gmail.com

**Keywords:** IGZO, TFT, oxygen flow, channel width, electrical property

## Abstract

The effects of various oxygen flows on indium gallium zinc oxide (IGZO) based thin-film transistors (TFTs) with different channel width sizes have been investigated. The IGZO nano-films exhibited amorphous phase while the bandgap energy and sheet resistance increased with increasing oxygen flow rate. The electrical characteristics were evaluated with different sizes in channel width using fixed channel length. The distributions in terms of threshold voltage and current on–off level along the different channel width sizes have been discussed thoroughly. The minimum distribution of threshold voltage was observed at an oxygen flow rate of 1 sccm. The TFT electrical properties have been achieved, using an oxygen flow rate of 1 sccm with 500 µm channel width, the threshold voltage, ratio of on-current to off-current, sub-threshold swing voltage and field effect mobility to be 0.54 V, 10^6^, 0.15 V/decade and 12.3 cm^2^/V·s, respectively. On the other hand, a larger channel width of 2000 µm could further improve the ratio of on-current to off-current and sub-threshold swing voltage to 10^7^ and 0.11 V/decade. The optimized combination of oxygen flow and channel width showed improved electrical characteristics for TFT applications.

## 1. Introduction

The flat panel display applications such as active-matrix liquid crystal displays (AMLCDs), active-matrix organic light-emitting diode (AMOLED) displays and electrophoretic displays are widely dependent on transparent conducting oxide (TCO) based thin-film transistor (TFT) as a switching element [1,2,3]. The TCOs are also useful for the next generation optoelectronic devices for the potential to produce high/low conductivity with high visual transparency at the same instant. The amorphous indium gallium zinc oxide (*a*-IGZO) has become one of the emerging TCO materials for TFT applications due to its high transmittance, low-cost processing at low temperature, and excellent surface quality [4,5]. It exhibits better performance than those based on hydrogenated amorphous silicon (*a*-Si:H), poly-Si and other TCO materials such as ZnO and IZO, in particular by electrical properties including high field-effect mobility, sharp sub-threshold swing, and high on/off current ratio characteristics [6,7,8]. Besides that, the *a*-IGZO film has been used in other applications such as an alignment layer for LCD and bio-sensor technology [9,10]. In addition, it contemplates an encouraging material for higher field effect mobility in amorphous phase which can be easily grown at lower temperature on Si, glass or flexible substrates using several deposition methods [11,12,13]. Among all, radio frequency (RF) sputtering has been used widely to achieve high-quality *a*-IGZO films. The other important issue is a suitable gate dielectric material for TFT devices. A simple, low-cost e-beam deposited SiO_2_ has been established as an alternative promising gate dielectric material to obtain high-performance *a*-IGZO TFT applications [14].

Inevitably, it is still desirable to optimize the process parameters such as argon flow, oxygen flow, deposition radio frequency power, substrate temperature, channel layer thickness and chamber pressure, etc. There have been several reports on the performances of *a*-IGZO TFTs to consider those types of process parameters during film formation [15,16,17]. Especially, the electrical properties of *a*-IGZO TFTs were influenced by the oxygen flow rate (OFR) during film growth immensely [18]. The physical properties of film could be affected by the absorption of oxygen molecules during film formation [19]. As an n-type material for doping, each oxygen vacancy generates two free electrons in the conductor band. The adsorbed oxygen at the surface can further influence the stability of IGZO TFTs [20]. The electron concentration decreased and would increase the resistivity in the oxide film with increased oxygen content, which influenced the electrical characteristics of the *a*-IGZO TFT [21]. On the other hand, the TFT performances depend not only on the process parameters but are also influenced by channel dimension with contact source-drain of *a*-IGZO TFT [22,23,24]. Generally, the TFT performances are not influenced too much by the variation of the channel width. The infant change of threshold voltage with a width of TFTs is considered as an infringe effect due to the instability of devices from the poor fabrication process. However, several researchers reported the noticeable variation of electrical properties with changing channel width of TFTs based on tunneling effect, heating effect and trap charge effect. For example, Lee et al. focused on the various channel width at a fixed length of TFTs using high-k dielectric material ZrO_2_ and obtained good performance and showed better stability of TFTs using argon ambient deposited *a*-IGZO film [25]. They also showed that the electrical performance changed slightly with a change in the size of the channel width of *a*-IGZO TFTs. In addition, Liu et al. investigated channel width-dependent threshold voltage variation in *a*-IGZO TFTs with different drain voltages on lower size to higher size channel width and have shown the noticeable variation of threshold voltage with channel width at low drain to source voltage [26]. Kwon et al. studied the performance of organic TFTs with variation in channel width and reported the large variation of threshold voltage and mobility with increased channel width [27]. Hatzopoulos et al. investigated the channel width-dependent electrical characteristics of bilayer amorphous and nano-crystalline silicon TFTs [28]. However, the conjugate effect of O_2_ flow rate and channel width on the electrical performances of *a*-IGZO TFTs has not been established. This type of study would be important to develop TFTs with different channel widths controlled by a common bottom gate structure deposited on the glass substrate at room temperature fabrication process. It is scientifically interesting to understand the basic device operation phenomena without a complicated high-temperature annealing process and the passivation layer. It is desirable to investigate the *a*-IGZO TFT performances at different channel dimensions using low-cost, easy processing e-beam deposited gate dielectrics fabricated on glass substrates for flat-panel display applications. It can be figured out with different O_2_ flow rates along the different sizes of channel width in *a*-IGZO TFTs with a possible mechanism. The variation of threshold voltage along the different size of channel width could be minimized with an optimized O_2_ flow rate. The different flow rates of O_2_ create more variation of electrical performances TFTs in threshold voltage, field mobility, the ratio of on-current to off-current and sub-threshold swing voltage along with changes of channel resistance, parasitic source-to-drain resistance, surface roughness and trap charge density, etc.

In this report, we focused on the influence of O_2_ flow rate on the electrical properties of different sizes of channel width *a*-IGZO TFTs. This study attempted to find the suitable oxygen flow during film growth for achieving uniform electrical characteristics from the low size to higher size width of TFTs with a possible mechanism. Thus, the fabrication of TFTs with different sizes of channel width would be more favorable to be employed in the display application. The variation of other performance parameters such as field effect mobility, the ratio of on-current to off-current, sub-threshold swing voltage, trap charge density was investigated in addition to a threshold voltage at different oxygen contents as well as different sizes of the channel width. It is therefore possible to obtain high-performance *a*-IGZO TFT with optimized O_2_ flow rate, the dimension of channel width, and argon flow rate using low-cost e-beam deposited SiO_2_ gate dielectric.

## 2. Materials and Methods

**Figure 1:**
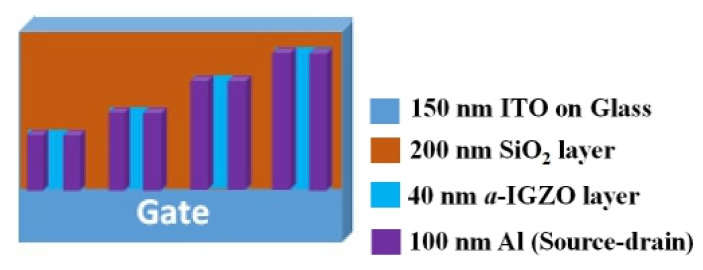
Schematic illustration of the device structure of amorphous indium gallium zinc oxide (*a*-IGZO) thin-film transistor (TFT) fabricated on a glass substrate.

**Figure 2:**
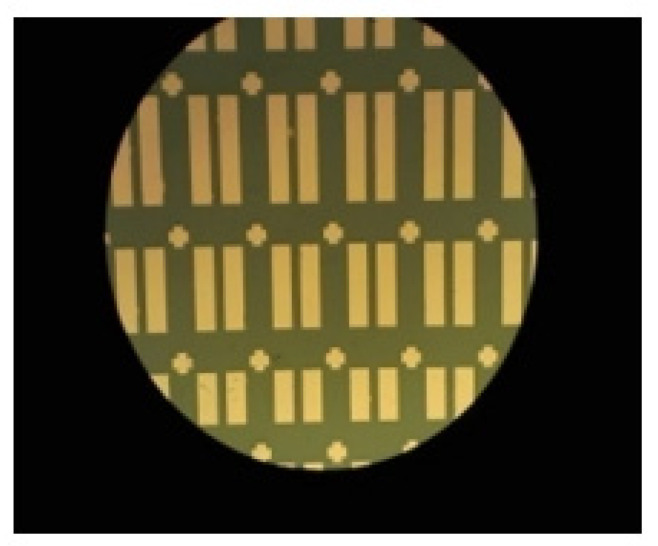
A micrograph of un-patterned *a*-IGZO TFT devices with different channel sizes.

The indium-tin oxide (ITO)-coated glass substrates had a sheet resistance of 15 Ω/sq. They were washed in acetone, alcohol and DI water, subsequently, before being dried by N_2_ gun and a hotplate. A vacuum tape was used to provide the common bottom gate of ITO. The experimental samples were deposited with gate dielectric SiO_2_ ~200 nm by an e-beam evaporator system. The e-beam chamber’s base vacuum pressure was 8 × 10^−6^ Torr. During the evaporation, it increased to about 10^−4^–10^−5^ Torr due to the evaporation of the depositing materials [29]. The chamber temperature was also slightly increased during the deposition to 23–30 ℃ due to the heating effect from the e-beam sources. The *a*-IGZO ~40 nm films were deposited by RF sputtering using the power of 70 W at 3 mTorr pressure under a fixed Ar flow rate of 30 sccm and different O_2_ flow rates, including 0, 1, 3 and 6 sccm. Then, 100 nm Al metallic film was deposited by a thermal evaporator system and was patterned to form the different sizes of TFT source/drain following photolithography and lift-off. The channel width was varied at 500 µm, 800 µm, 1500 µm and 2000 µm. The channel length was fixed at 200 µm. On the other hand, SiO_2_ was deposited on p + Si at the same time for capacitance measurement and *a*-IGZO film grown on clean bare glass for the investigation of physical characteristics of films. A schematic illustration of the device structure is shown in Figure 1. A micrograph of the un-patterned common gate with different channel width based *a*-IGZO TFTs is displayed in Figure 2. The optical transmittance characteristics (Jasco, ISN-723, Tokyo, Japan), X-ray diffraction (XRD, Bruker, D2 Phaser, Billerica, MA, USA) analysis, and X-ray photoelectron spectroscopy (XPS, VG Scientific Microlab 350, Waltham, MA, USA) were carried out. The sheet resistance of *a*-IGZO film was measured using a Hall-effect automatic measurement system (KeithLink Tech, Taipei, Taiwan). For quality assurance, the films were grown on cleaned bare glass at the same time when preparing for the TFT samples. In addition, the TFT devices’ electrical properties were evaluated by a semiconductor parameter analyzer (Agilent, B1500A, Santa Clara, CA, USA).

## 3. Results and Discussion

**Figure 3:**
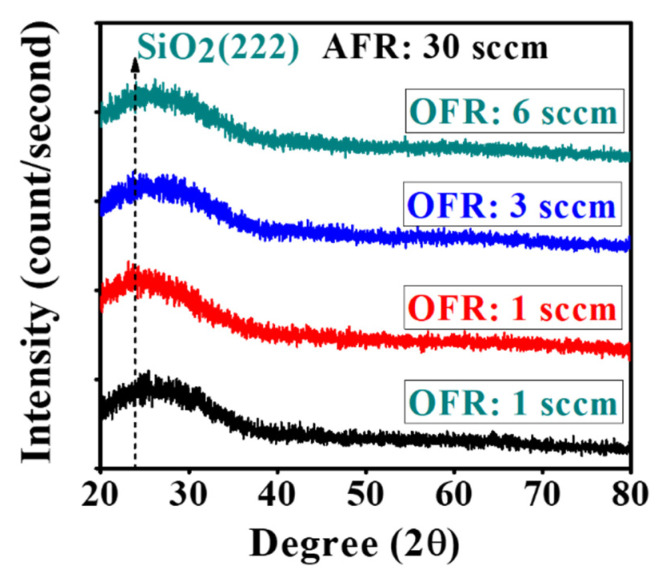
The amorphous phase of the as-deposited IGZO films at different O_2_ flow rates was confirmed by XRD analysis.

To investigate the effects of O_2_ flow rate during sputtering of IGZO film, the film microstructure was characterized by X-ray diffraction analysis. The amorphous structure was confirmed by XRD analysis at different O_2_ flow rates at constant argon flow. The amorphous structure remained unchanged with the increased oxygen flow during film growth, as shown in Figure 3. The notable halo crest occurs at 23° from SiO_2_ of the glass substrate but there was no individual sharp diffraction peak for crystalline structure because of the irregular arrangement of atoms inside of layer. The physical structure of *a*-IGZO films at different oxygen contents has not been well-justified because of the amorphous phase. Nevertheless, the crystalline properties can be achieved at higher O_2_ flow with high-temperature annealing in vacuum ambient [30].

**Figure 4:**
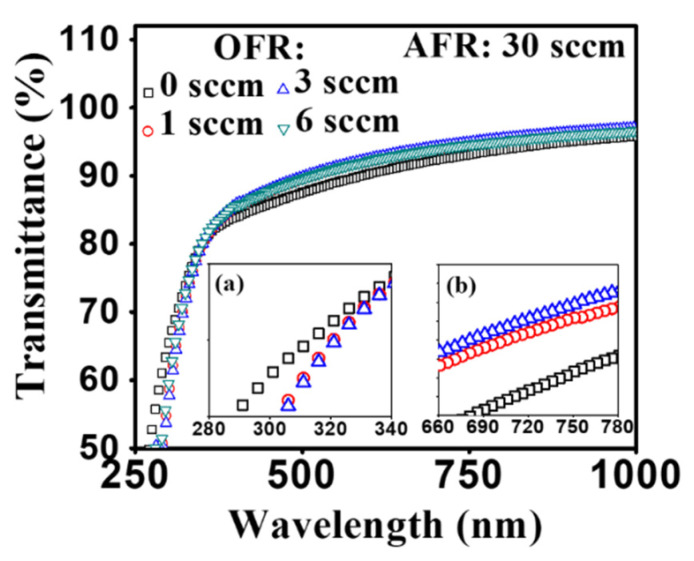
Optical transmission spectra of radio frequency (RF) sputtered *a*-IGZO films on clean bare glass with different O_2_ flow rates during film growth. Subfigures are shown for the variation of transmittance at (**a**) shorter and (**b**) longer wavelength range.

The optical transmittance spectra at different oxygen flow rates of the as-deposited *a*-IGZO films on the clean bare glass are shown in Figure 4. It has appeared that the transmittance is higher than 90% in the visible and near-infrared range (wavelength 450 nm to 1000 nm) for the RF sputtered films under the various O_2_ flow rates. The transmittance increased with an increased O_2_ flow rate from 0 sccm to 3 sccm at the longer wavelength region due to improved internal film quality with a decreased defect. The oxygen ion could easily escape during the deposition. However, the transmittance at the highest O_2_ flow rate of 6 sccm dropped slightly, likely caused by the defects in the nanostructure of film due to the high density of oxygen content. A sharp absorption occurred near 350 nm only for the oxygen-based samples because of the strong electronic band transitions of carriers. It is also shown that at lower than 370 nm wavelength region the transmittance drops sharply for all the IGZO films (inset (a)). This suggests improved film quality using oxygen flow in conjunction with the argon flow process. The bandgap energy of each film can be calculated using the plot method by the equation in [31]:

*αhν* = *k* (*hν* − *E*_g_)^1/2^, (1)

where *α*, *h* and *ν* represent the absorption coefficient of *a*-IGZO film, Planck constant and the radiation frequency, and *k* is a constant. The absorption coefficient is determined using the following equation [32]

*α* = [ln(1/*T*)]/*d*, (2)

where *T* is the measured transmittance and *d* is the film thickness. The optical band gap (*E*g) can be obtained by extrapolating the straight-line portion of (*αhν*)^2^ vs. *hν* plots to the energy axis. The bandgap energy extends from 3.47 eV to 3.55 eV with the increase in O_2_ flow rate from 0 to 6 sccm. This phenomenon is likely caused by a change in carrier concentration [33,34].

**Figure 5:**
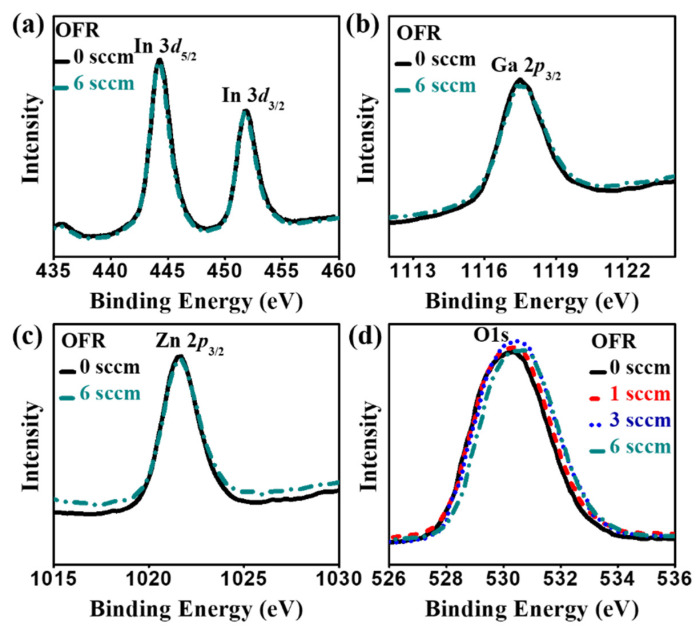
The XPS analysis of the as-deposited *a*-IGZO films with the different oxygen flow rates: (**a**) In 3d_5/2_ (**b**) Ga 2p_3/2_ (**c**) Zn 2p_3/2_ and (**d**) O1s (solid, dash, dot and dash–dot lines are indicated for OFR at 0 sccm, 1 sccm, 3 sccm and 6 sccm, respectively).

The XPS analysis was carried out for further physical characteristics of the as-deposited *a*-IGZO films, and the results are shown in Figure 5. The variation of the binding energy of In 3d_5/2_, Ga 2p_3/2_ and Zn 2p_3/2_ are not so much affected as compared with the O1s state by the change in oxygen flow rate. The chemical shift of O1s is more prominent than the other electronic states because of the insensitive nature of In, Ga and Zn at the normal environment. The binding energy values at the peak intensity of O1s state with an oxygen flow rate of 0 sccm, 1 sccm, 3 sccm and 6 sccm are 530.2 eV, 530.4 eV, 530.5 eV and 530.7 eV, respectively. The binding energy values at peak intensity for all the elements with different OFR are shown in Table 1. The enhancement in binding energy suggested that the carrier concentration of film was altered when more oxygen content was introduced in the film. The drop in intensity at a high O_2_ flow rate of 6 sccm agrees well with the earlier results in enhanced defect properties with decreased transmittance. Yao et al. revealed that the electron concentration decreased with increased oxygen flow rate while the atom ratios of ZnO, Ga_2_O_3_, and In_2_O_3_ remained almost the same [35]. Lee et al. found that the value of Ga/(In + Ga + Zn) and Zn/(In + Ga + Zn) ratio was increased and In/(In + Ga + Zn) decreased with increasing oxygen flow rate [36]. The noticeable variation of the binding energy of In 3d5/2, Ga 2p3/2 and Zn 2p3/2 could be changed with various RF power during a-IGZO film growth [37].

**Figure 6:**
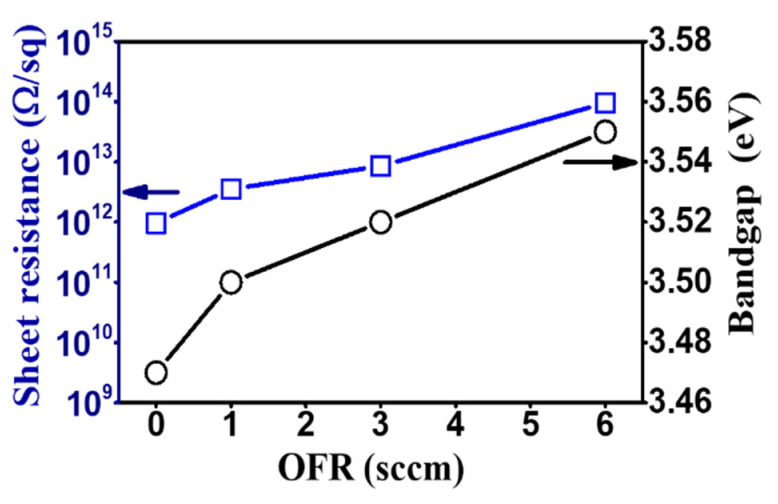
The variation of sheet resistance and bandgap energy of the as-deposited *a*-IGZO films with various oxygen flow rates.

In order to change the parasitic resistance of source to drain with different width channel of *a*-IGZO TFTs, the surface sheet resistance was measured to understand the surface resistance properties of the as-deposited *a*-IGZO films at different O_2_ flow rates. The sheet resistance values of the *a*-IGZO surface are 9.64 × 10^11^ Ω/sq, 3.54 × 10^12^ Ω/sq, 8.53 × 10^12^ Ω/sq and 9.54 × 10^13^ Ω/sq for the various O_2_ flow rate of 0 sccm, 1 sccm, 3 sccm and 6 sccm, respectively. The sheet resistance increasing tendency revealed the high surface roughness as well as decreased carrier concentration of film due to the higher density of oxygen content plasma. The sheet resistances were evaluated to set up the possible correlation with different sizes of channel width at each O_2_ flow rate. The variation of sheet resistance and bandgap energy with different oxygen flow are plotted together in Figure 6.

**Figure 7:**
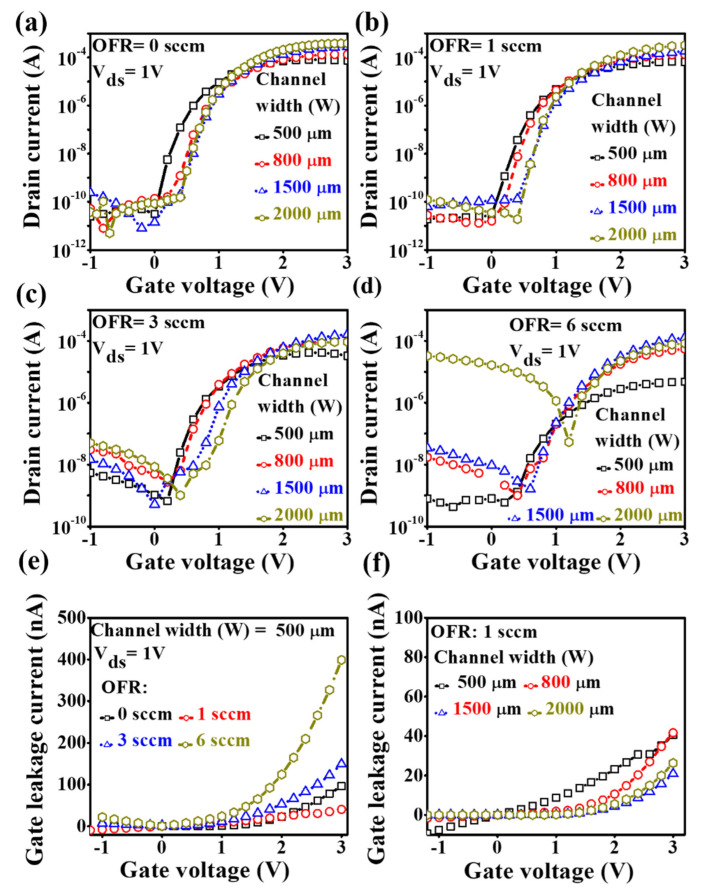
Typical transfer characteristics of *a*-IGZO based TFT with oxygen flow rate of (**a**) 0 sccm, (**b**) 1 sccm, (**c**) 3 sccm and (**d**) 6 sccm. The fixed drain voltage is 1 V, gate voltage sweeping from −1 V to +3 V (the open square with solid line, open circle with dash line, open triangle with dot and open hexagon with dash–dot line represent the transfer characteristics for channel width 500 µm, 800 µm, 1500 µm and 2000 µm, respectively). The effects of (**e**) OFR and (**f**) channel width (W) on gate leakage current are presented.

The influence of O_2_ flow rate is substantial by channel width variation. Figure 7 displays the transfer characteristics of *a*-IGZO TFT with the different O_2_ flow rate at 0 sccm, 1 sccm, 3 sccm and 6 sccm. The drain voltage was fixed at 1 V, and the swing gate voltage was varied from −1 V to +3 V. In general, the transfer characteristics showed that a minimum distribution in terms of threshold voltage variation, on-current and off-current level along the increasing channel width, was observed from Figure 7b using the oxygen flow rate of 1 sccm. On the other hand, the maximum distribution was found from Figure 7d with the highest O_2_ flow rate at 6 sccm. It should be noted that the off-current level increased up at the beginning of negative voltage through the lower size to higher size devices, especially at the −1 to 0 V negative bias. Also, there has been smoother characteristics respect to off-current level, and on-voltage from sweep gate voltage −1 V to 3 V achieved only at oxygen flow rate of 1 sccm rather than the other flow rates. In order to understand the impact on the electrical prosperities by the process parameter of various O_2_ flow rate and physical dimension of channel width, we further characterized each transfer characteristic curve to obtain field-effect mobility (*µ*_fet_), threshold voltage (*V*_th_), sub-threshold swing voltage (*SS*) and the ratio of on current to off current (*I*_on_/*I*_off_). Consequently, the reason for the dissimilarity of each parameter would be discussed on the mechanism due to oxygen flow treatment and channel width. After that, both effects would be combined to show how they influence together on electrical characteristics of *a*-IGZO TFTs. Furthermore, the effects of OFR and channel width on gate leakage current are shown in Figure 7e,f. The leakage current increased substantially with increasing OFR for the channel width of 500 μm, which could impact the device’s performances. On the other hand, the effect of channel width on gate leakage current at OFR of 1 sccm could be considerably smaller.

The field-effect mobility can be extracted using the following equation:

(3) $${µ}_{\mathrm{fet}}\;=\;\frac{g_{m}\;\times \;L}{C_{\mathrm{ox}}\;\times \;W\;\times \;V_{\mathrm{ds}}},$$

where *g*_m_ is the transconductance that is defined by the derivative of drain current with respect to gate voltage, *L* is the length of the channel, *W* is the width of the channel and *C*_ox_ is gate oxide capacitance per unit area which was obtained at 4 nF/cm^2^ by C-V measurement of MOS capacitor using the same thickness and maintained same deposition condition of SiO_2_. The obtained field effect mobility without O_2_ flow-based sample was 11.9 cm^2^/V·s, 9.4 cm^2^/V·s, 8.3 cm^2^/V·s and 7.4 cm^2^/V·s for the corresponding channel widths of 500 µm, 800 µm, 1500 µm and 2000 µm, respectively. The mobility was slightly improved for each size of channel width based TFTs when introduced with small amount of oxygen flow rate at 1 sccm and the values became 12.3 cm^2^/V·s, 10.8 cm^2^/V·s, 9.4 cm^2^/V·s and 9.1 cm^2^/V·s. However, the mobility has become lower at the higher oxygen flow rates at 3 sccm and 6 sccm, with a maintained tendency of decreased associates along with increased channel width size. The calculated mobility results for all sizes of channel width at different oxygen flow rates are presented in Table 2, Table 3, Table 4 and Table 5. The field effect mobility decreased with increasing O_2_ flow rate for each size of channel width because of the increased defects. This trend is in good agreement with the reported results based on the oxygen flow rate study [38]. It is also effectively degraded with increasing channel width at each fixed oxygen flow rate due to the increased surface to volume ratio of the channel surface with different contact size of channel width-based source-drain. However, the mobility could be increased with increasing channel width due to the decrease of the source-drain contact resistance at a fixed ratio of W/L [39]. The mobility could be improved using the little oxygen content of plasma during the deposition. The fluctuations of mobility along the channel width were also suppressed at the lower oxygen flow rate. These changes of mobility may arise from the dimension effect of TFTs rather than just oxygen flow rate. The high oxygen flow rate at 6 sccm greatly influenced the mobility of TFTs and resulted in very low mobility for all types of channel width because of not only higher defects and decreased carrier concentration but also the increased parasitic effect due to higher sheet resistance with increasing sizes of source-drain. These results also suggest that field effect mobility with the various channel width can be controlled using the appropriate oxygen flow rate in plasma during film growth.

The threshold voltage (*V*_th_) is calculated from each transfer characteristic curve by extra-plotting of *I*_ds_^1/2^ vs. *V*_gs_, and the calculated values are also shown in Table 2, Table 3, Table 4 and Table 5. The threshold voltages for the pure argon based samples are 0.41 V, 0.65 V, 0.89 V and 0.90 V, corresponding to the channel width of 500 µm, 800 µm, 1500 µm and 2000 µm, respectively. The threshold voltage differs by 0.49 V from the lower size to the higher size channel width. On the other hand, the threshold voltage for the oxygen flow rate at 1 sccm based samples are 0.54 V, 0.66 V, 0.77 V and 0.86 V, with a corresponding channel width of 500 µm, 800 µm, 1500 µm and 2000 µm, respectively. It has been noted that a hysteresis analysis by taking a double sweep could help to extract information on gate insulator and interface quality [40,41]. The variation of the threshold voltage is about only 0.32 V from the lower to higher size channel width. The threshold voltage was increased with increasing channel width, while the variation values from lower to higher size channel width at an oxygen flow rate of 3 sccm and 6 sccm are 0.43 V and 0.52 V. It has been suggested that the threshold voltage is small for the smaller channel width of the device and the threshold voltage is increased with increasing channel width at each O_2_ flow rate. It increased as well with an increased O_2_ flow rate at a fixed channel width. The threshold voltage change with channel width has been well-maintained with respect to the O_2_ flow rate fixed at 1 sccm. The effects of both oxygen content and dimension of channel width played some important roles. It became more irregular when the oxygen flow increased to a higher rate at 6 sccm. The higher size devices associated with 1500 µm and 2000 µm squeeze the threshold voltage at an oxygen flow rate of 1 sccm even more than the samples with no oxygen flow. The results indicate that the proper amount of oxygen flow can be helpful to improve the uniformity of threshold voltage and to avoid the sort of channel effect of TFT from low order to high order device size for TFTs with different sizes of the channel width. The distribution of threshold voltage would be also affected by the contact of source-drain on channel surface due to sheet resistance. The total volume to surface ratio changes with channel width as the length remains constant.

Sub-threshold swing voltage (*SS*) is one of the key factors to characterize transistor performance for high speed and low power operation in low voltage portable devices. It is determined from the slope of log *I*_ds_ versus *V*_gs_ curve and the corresponding equation is

(4) $$SS\;=\;\frac{d\;V_{\mathrm{gs}}}{d\left(\log\;I_{\mathrm{ds}}\right)}.$$

The evaluated values of *SS* at pure argon based samples are 0.18 V/decade, 0.22 V/decade, 0.16 V/decade and 0.13 V/decade, and at an oxygen flow rate of 1 sccm they are 0.15 V/decade, 0.17 V/decade, 0.13 V/decade and 0.11 V/decade with the corresponding channel width of 500 µm, 800 µm, 1500 µm and 2000 µm, respectively. The very small *SS* is obtained at about 0.11 V/decade from the biggest size of the channel width of 2000 µm at an O_2_ flow rate of 1 sccm and all the other data are also shown in Table 2, Table 3, Table 4 and Table 5. The results have indicated that the uniformity of *SS* can be obtained with a small amount of oxygen flow during film deposition. The *SS* values are somewhat increased when much more oxygen is introduced to the channel deposition. The SS values are shown lower along with the sizes of channel width with the low oxygen flow rate of 1 sccm because of the smoother surface of channel material with respect to the other samples. It is well known that small SS values can lead to higher mobility and good stability of TFTs. Therefore, the optimized O_2_ flow rate and channel width would be associated with 1 sccm and 2000 µm, respectively. The small *SS* values are also attributed to the lower number of interfacial trap charge densities in the interfacial surface of the channel and gate dielectric. It would be expected with a lower number of interfacial charges produced at an O_2_ flow rate of 1 sccm because of lower values of *SS*. The higher number of interfacial charge densities may play a more important role to increase the off-current level due to higher leakage current with increased O_2_ flow rate, and also increased channel width.

The TFT performances are very much dependent on the ratio of on-current to the off-current (*I*_on_/*I*_Off_) and all the evaluated values with channel width and O_2_ flow rate are shown in Table 2, Table 3, Table 4 and Table 5. It is significantly changed when increased O_2_ flow rate on the *a*-IGZO film deposition. Even without oxygen inlet, the *I*_on_/*I*_Off_ achieved higher than 10^7^ for the larger width of the channel but remarkably decreased at the higher O_2_ flow rates of 3 and 6 sccm. It is shown about 10^3^–10^4^ for the higher channel width and even so for the smaller sizes of channel width-based *a*-IGZO TFTs. The on-current level was increased with increasing channel width at an oxygen flow rate of 0 sccm and 1 sccm because of the increased current in the channel. The phenomena are in good agreement with the reported results by Liu et al. [26]. The distribution in both on- and off-current levels was achieved along the different channel width sizes when more oxygen was introduced during the film formation. The off-current level at the initial negative voltage is increased with the increasing channel width from lower sizes to higher sizes at each fixed O_2_ flow rate. The higher channel width thus produced more leakage current that increased the off-current level. This phenomenon disturbed strongly at the higher oxygen flow rate at 6 sccm. The off-current level reached above 10^−5^ order for the largest channel width of 2000 µm based TFTs. This disturbance might be caused by the increased self-heating effect from the higher surface roughness of the channel [42]. The corresponding threshold voltage was also increased to 1.16 V. On the other hand, the on-current level for the higher size devices behaved normally like the higher on-current level corresponding to the higher size devices at an oxygen flow of 0 and 1 sccm. This effect became prohibited for the higher oxygen flow rate samples.

**Figure 8:**
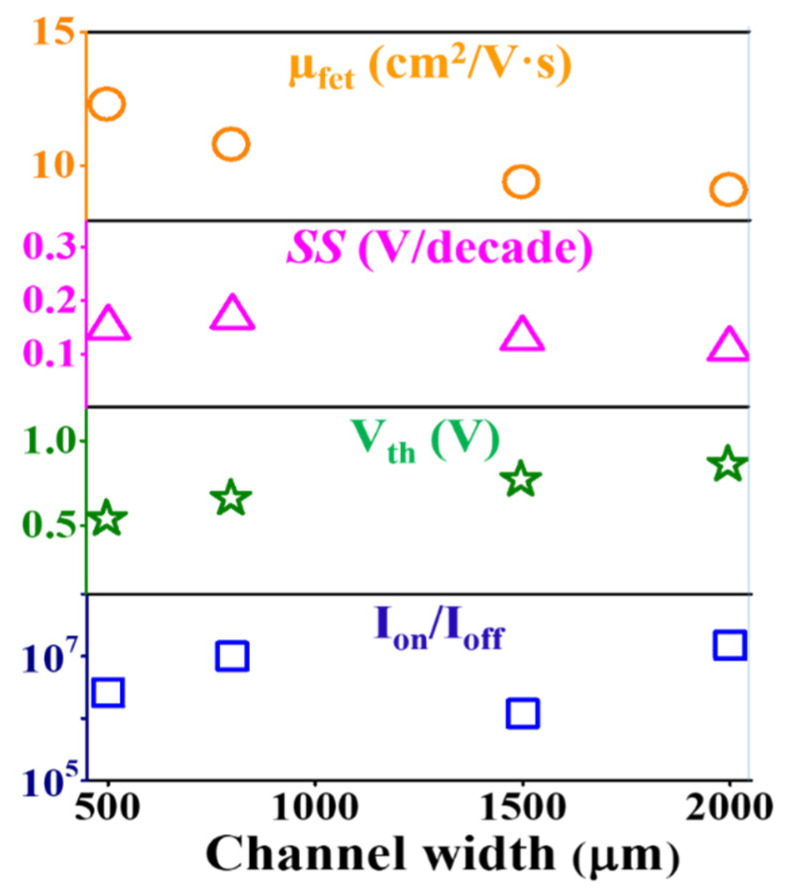
The field effect mobility (*µ*_fet_), sub-threshold swing voltage (*SS*), threshold voltage (*V*_th_), and ratio of on-current to off-current (*I*_on_/*I*_off_) with different sizes of channel width at an oxygen flow rate of 1 sccm.

**Figure 9:**
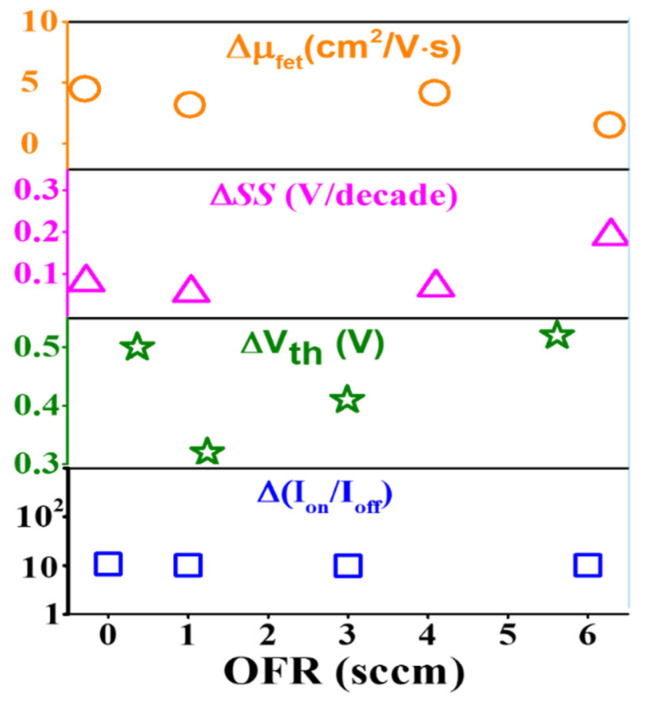
The variation (∆) of each electrical parameter from the lower to higher sizes channel width with the various O_2_ flow rates.

In the discussion, the *a*-IGZO TFTs electrical performance parameters are dependent upon the O_2_ flow rate during sputtering. The transfer characteristics from the lower to higher size channel width at different O_2_ flow rate showed lots of dissimilarities, such as distribution of on- and off-current level. The variation of the off-current level along O_2_ flow rate followed a more-or-less linear fashion. The worst distribution was observed at the high O_2_ flow rate at 6 sccm. The leakage current increased above 10^−6^ at higher sizes of channel width-based *a*-IGZO TFTs. It should be noted that the leakage current variation at negative gate voltage 1 V along the channel width depends on the oxygen flow rate. It is varied only by one order at O_2_ flow rate of 1 sccm and is increased the order at 0 sccm as well as the higher flow rates of 3 sccm and 6 sccm. The variations are shown in the same manner for each channel width-based TFTs. In this report, the threshold voltage was also increased with increasing O_2_ flow rate due to the higher resistivity of the film. The subthreshold swing voltages increased with increasing O_2_ flow rate because of the increased defects. The off-current level is increased with increasing oxygen flow rate due to the increased defect in the interfacial region and the lower density of oxygen vacancy suppresses the carrier concentration in the film. In addition, the channel width dependent field effect mobility, threshold voltage, subthreshold swing voltage and ratio of on-current to the off-current at a fixed O_2_ flow rate of 1 sccm are plotted together, as shown in Figure 8. The variation of threshold voltage with channel width is short of uniformity as compared with conventional devices. However, the increased tendency of mobility and on-current with increasing channel width has been reported by Lee et al. without considering the oxygen flow effect on the *a*-IGZO surface [24]. Nevertheless, the process parameter of O_2_ flow rate changed the sheet resistance of the channel surface which could increase the parasitic effect of source-drain on channel material. This effect gradually increased as the channel width of the *a*-IGZO TFT was increased [22]. Therefore, the O_2_ flow rate effect on the TFTs with different sizes of channel width lead to large variation without considering the optimization of O_2_ flow rate during the deposition of *a*-IGZO films. The source to drain resistance could influence the electrical performances of *a*-IGZO TFT and as a result to have achieved the short channel effect due to the lack of ohmic contact. This affected the voltage drop at the source and drain contacts. In addition, the water and oxygen molecules in the atmosphere can also affect the device performance of the oxide semiconductor-based TFTs, due to the lack of a passivation layer or cap layer [20]. A suitable passivation layer could be thus employed to improve the uniformity of the *a*-IGZO TFTs. The variation (∆) of each of the electrical parameters from low value to high value with the various O_2_ flow rates are plotted together, as shown in Figure 9. In addition, the e-beam deposited SiO_2_ is a promising gate dielectric at a low drain voltage of 1 V.

The phenomena regarding changing performance parameters of *a*-IGZO TFTs at the higher fixed drain voltage of 2~5 V exhibited a similar trend. However, at drain voltage higher than 6 V, the performances were degraded due to the intrinsic characteristics of the as-deposited gate dielectric SiO_2_. Further annealing and/or plasma treatment can be developed to improve the quality of the SiO_2_ dielectric film.

**Figure 10:**
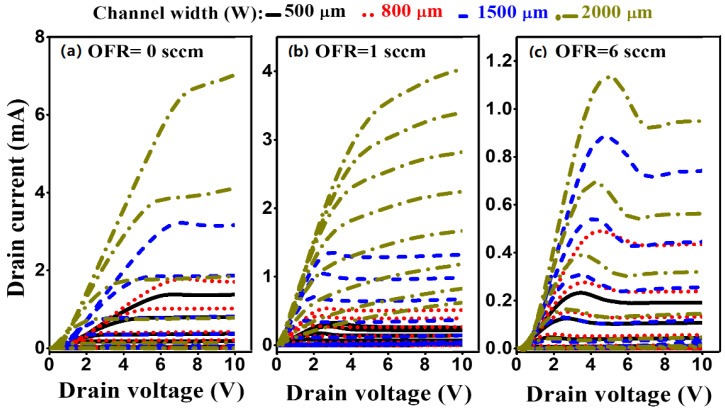
The output characteristics of *a*-IGZO TFT at an oxygen flow rate of (**a**) 0 sccm, (**b**) 1 sccm and (**c**) 6 sccm. The gate voltage varied −1 V to 3 V with an increased step voltage of 0.5 V. (Solid, dot, dash and dot–dash lines represented for channel width 500 µm, 800 µm, 1500 µm and 2000 µm, respectively).

The output characteristics of the *a*-IGZO TFT are shown in Figure 10 for the devices from O_2_ flow rate of 0 sccm, 1 sccm and 6 sccm for the lower size to higher size channel width. All the output characteristics exhibited positive drain current at zero gate voltage because of carrier transportation of channel by the drain to source voltage. Again, the output characteristics supported the better ohmic performance of *a*-IGZO TFT at an oxygen flow rate of 1 sccm than the other OFR samples. Three different output characteristics were shown with saturation region, saturation current and pinch of voltage. On the other hand, the hump effect increased with increasing O_2_ flow rate. The effect might be originated from structural defects which act as traps for the charge carriers under bias. Thus, higher OFR introduced higher structural defects and decreased the ohmic contact properties of metal on the *a*-IGZO surface, especially for large channel width. The stress-induced hump phenomenon was also observed by Choi and Han for wide channel width (W > 100 μm) [43]. This problem could be avoided by employing the post-fabrication annealing process. However, the characteristics have been shown with excellent n-type enhancement mode with low drain current at zero gate voltage. In addition, the output characteristics revealed that the ohmic contact could be affected by sheet resistance with unsuitable O_2_ content in the a-IGZO film. The stability and better ohmic contact could be further improved with a surface treatment such as annealing, plasma process, or even by replacing the metal of source-drain contact.

## 4. Conclusions

In conclusion, O_2_ flow rate is one of the key factors for obtaining better performances, especially for TFTs with different sizes of the channel width. The results showed that the suitable channel width at an optimized oxygen flow rate could produce high-performance *a*-IGZO TFTs. However, the very high O_2_ flow rate could degrade the performance of TFT, in particular *I*_on_/*I*_off_ ratio as compared with the small amount of O_2_ in the irrespective channel width. The small amount O_2_ flow rate would be sufficient to obtain high performances and also to exhibit less variation with channel width in TFTs. This study has paved the way to minimize the variation of threshold along the different dimension TFTs usingthe single optimized oxygen flow rate. The optimized TFT electrical properties were achieved at the oxygen flow rate of 1 sccm with 500 µm channel width. The threshold voltage, the ratio of on-current to off-current, sub-threshold swing voltage and field effect mobility were 0.54 V, 10^6^, 0.15 V/decade and 12.3 cm^2^/V·s, respectively. The mobility decreased with increasing oxygen flow rate from 1 to 6 sccm by ~55%. The threshold voltage increased by ~16% at the constant channel width of 500 µm. The better ohmic contact behavior and high performance TFTs can be achieved while considering both the effects of O_2_ flow rate and the channel width design for the future TFT applications.

## Figures and Tables

**Table 1:** The binding energy values at peak intensity of In 3d_3/2_, Ga 2p_3/2_, Zn 2p_3/2_, and O 1s electronic state with different oxygen flow rate (OFR), along with the corresponding standard values.

Element		In3d_3/2_	Ga2p_3/2_	Zn 2p_3/2_	O1s
Standard value		451.3	1117.4	1021.5	531.4
OFR	0	451.8	1117.5	1021.7	530.2
	1	451.8	1117.5	1021.7	530.4
	3	451.8	1117.5	1021.7	530.5
	6	451.8	1117.5	1021.6	530.7

**Table 2:** The electrical performance parameters of *a*-IGZO TFT with different sizes of channel width at an oxygen flow rate of 0 sccm.

Channel Width (µm)	*V*_th_ (V)	*I*_on_/*I*_off_	*SS* (V/decade)	*µ*_fet_ (cm^2^/V⋅s)
500	0.41	2.2 × 10^6^	0.18	11.9
800	0.65	1.6 × 10^6^	0.22	9.4
1500	0.89	3.0 × 10^7^	0.16	8.3
2000	0.90	1.1 × 10^7^	0.13	7.4

**Table 3:** The electrical performance parameters of *a*-IGZO TFT with different sizes of channel width at an oxygen flow rate of 1 sccm.

Channel Width (µm)	*V*_th_ (V)	*I*_on_/*I*_off_	*SS* (V/decade)	*µ*_fet_ (cm^2^/V⋅s)
500	0.54	2.6 × 10^6^	0.15	12.3
800	0.66	1.0 × 10^7^	0.17	10.8
1500	0.77	1.2 × 10^6^	0.13	9.4
2000	0.86	1.5 × 10^7^	0.11	9.1

**Table 4:** The electrical performance parameters of *a*-IGZO TFT with different sizes of channel width at an oxygen flow rate of 3 sccm.

Channel Width (µm)	*V*_th_ (V)	*I*_on_/*I*_off_	*SS* (V/decade)	*µ*_fet_ (cm^2^/V⋅s)
500	0.58	5.9 × 10^5^	0.23	10.2
800	0.69	3.4 × 10^5^	0.27	8.8
1500	0.98	2.7 × 10^5^	0.21	7.6
2000	1.0	8.2 × 10^4^	0.20	5.8

**Table 5:** The electrical performance parameters of *a*-IGZO TFT with different sizes of channel width at an oxygen flow rate of 6 sccm.

Channel Width (µm)	*V*_th_ (V)	*I*_on_/*I*_off_	*SS* (V/decade)	*µ*_fet_ (cm^2^/V⋅s)
500	0.64	5.9 × 10^3^	0.35	5.5
800	0.94	4.2 × 10^4^	0.26	4.6
1500	0.98	7.1 × 10^4^	0.25	3.9
2000	1.16	1.2 × 10^3^	0.44	-
